# Nuclear Deformation and Stiffness-Dependent Traction Force Generation Dictate the Migration of Cells under Confinement

**DOI:** 10.1021/acsami.5c03048

**Published:** 2025-04-15

**Authors:** Zheng Wang, Feng Xu, Di Wu, Wei Huang, Zhiqin Chu, Yuan Lin

**Affiliations:** † Department of Mechanical Engineering, The University of Hong Kong, Central and Western District, Hong Kong SAR 999077, China; ‡ Department of Electrical and Electronic Engineering, The University of Hong Kong, Central and Western District, Hong Kong SAR 999077, China; § Advanced Biomedical Instrumentation Centre, Hong Kong Science Park, Shatin, New Territories, Hong Kong SAR 999077, China

**Keywords:** cell migration, confinement, hydrogel printing, nuclear deformation, ECM stiffness, theoretical model

## Abstract

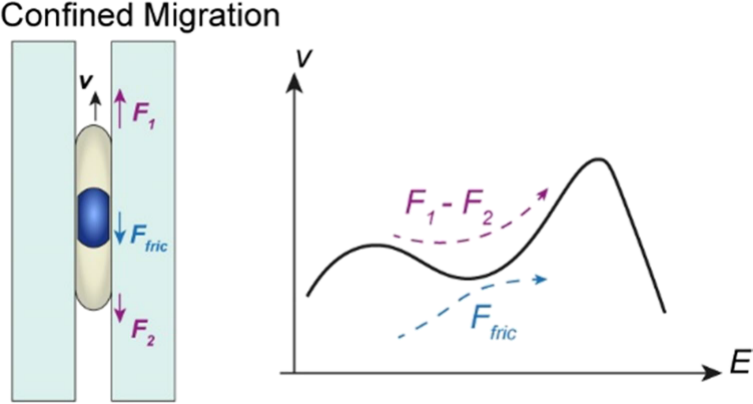

Cells need to migrate through confined spaces during processes such as embryo development and cancer metastasis. However, the fundamental question of how confinement size and surrounding rigidity collectively regulate the migration capability of cells remains unclear. Here, by utilizing maskless photolithography with a digital micromirror device (DMD), a microchannel with precisely controlled width and wall stiffness (similar to those exhibited by natural tissues) is fabricated. We find that increasing the rigidity of the confining wall leads to a more reduced nuclear volume but has no detectable influence on the myosin expression level in the cells. More interestingly, a biphasic trend of the cell speed is observed, with the migration velocity reaching its minimum at an intermediate wall rigidity of ∼10 kPa. A motor-clutch-based pulling race model is then proposed, which suggests that such biphasic dependence is due to the fact that a very soft channel wall will result in small deformation of the nucleus and consequently reduced cell-wall friction, while larger myosin-based crawling force can be triggered by a stiff confining boundary, both leading to a relatively high migration speed. These findings could provide critical insights into novel strategies for controlling the movement of cells and the design of high-performance biological materials.

## 1 Introduction

Cell migration is critical in processes such as wound healing, tumor development, and morphogenesis. In metastasis, for example, cancer cells escape from the primary tumor, enter the circulation system, and eventually rethrive at a distant site.^1^ Given that micropores and tunnel-like microtracks naturally exist in tissues/extracellular matrices (ECMs)^2,3^ or can be produced by the cancer-associated fibroblasts with metalloproteinases,^4,5^ tumor cells likely need to migrate through these tight spaces during this process. In addition, once entering the blood vessels, the metastasizing cells are able to squeeze through the micron-sized capillaries.^6,7^ However, the fundamental question of how the migration capability of cells is influenced by spatial confinement from their deformable surrounding environment remains unclear.

**Figure 1:**
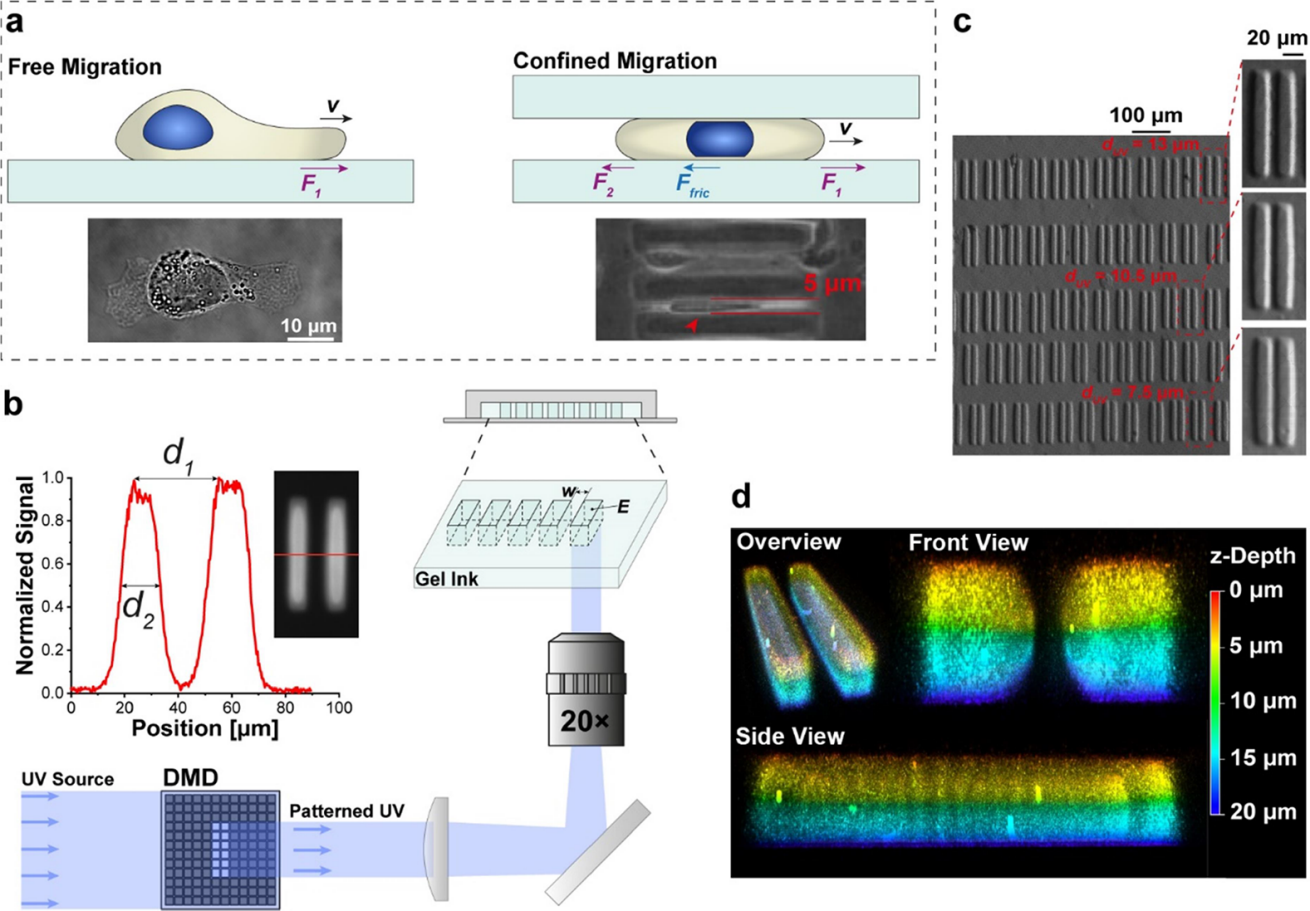
Modeling confined cell migration in vitro via photoprinted hydrogel microtunnels with precisely controlled geometry and stiffness. (a) Migrating speed (*v*) of cells on a free surface is largely dominated by the crawling force (*F*_1_) generated at the leading edge of the cell. In contrast, significant frictional force (*F*_fric_) could be generated between the deformed cell body/nucleus and the surroundings, which slows down the movement of cells. Actual images of cells migrating on a free surface or in a confined tunnel (indicated by the red arrow) are also presented. (b) Confining environment could be generated via an array of hydrogel microtunnels printed on a microchip with the DMD-based UV-patterning system. Specifically, by controlling the pattern geometry and exposure time, tunnels with designed modulus (*E*) and width (*w* = *d*_1_ – *d*_2_) (see the inset) and wall thickness (*d*_2_) can be fabricated. (c) Actual images of printed tunnel arrays using different UV patterns. Here, *d*_uv_ represents the width of the microtunnel pattern. (d) Confocal images showing the 3D structure of the printed hydrogel tunnel.

In particular, although it is widely known that many cells prefer to migrate toward the stiffer regions of a 2D substrate,^8^ such durotaxis behavior becomes questionable when cells move within a confinement environment that could impose large deformation on the cell and therefore provide an elevated frictional force against its movement (Figure 1a). Indeed, a significantly reduced crawling speed of cancer cells was observed in stiff confining microtunnels.^9,10^ Furthermore, due to the rigidity-dependent friction between the confining environment and motile cells, as well as confinement-triggered cellular actions, the widely accepted view that maximum cell speed will be achieved at intermediate substrate rigidity (reflecting the fact that little traction forces can be generated through molecular clutches connecting the cell to the substrate when the ECM is either very soft or very stiff^11^) could also break down (Figure 1a). For example, recent evidence has shown that a severely deformed cell nucleus will expose the folded domains of the nuclear membrane, activate actomyosin contraction, and eventually lead to elevated cell motility in a highly confined microenvironment.^12,13^ It must be pointed out that the confining materials used in these studies are very stiff. In reality, however, cells will most likely move in an environment with rigidity ranging from a few to a few tens of kPa (like that for normal or fibrotic tissues in cancer patients^14^), which is comparable to the modulus of the cell nucleus (typically of the order of several kPa). Therefore, instead of inducing large nuclear distortion and triggering myosin contraction, the confining boundary itself may deform under such circumstances, resulting in a much more complicated regulation of the migration of cells.

Here, we report a combined experimental and modeling study to address these unsettling issues. Specifically, by using gelatin methacryloyl (GelMA) that can be cross-linked under ultraviolet (UV) irradiation and DMD-based maskless photolithography (Figure S1a,b), microsized tunnels with precisely controlled width and wall rigidity were fabricated.^15−17^ The nuclear morphology, myosin activity, and migration velocity of cells within these highly confined tunnels were then systematically examined. In addition, by considering the friction between the cell and the tunnel wall and the crawling force generated by actomyosin contraction, a motor-clutch-based pulling race model was also developed to explain the observed biphasic dependence of cell speed on the stiffness of the confining boundary.

## 2 Experimental Section

### 2.1 Preparation of GelMA Precursor

GelMA polymer was synthesized using the previous method (Figure S5).^18^ Briefly, 3.5 g of gelatin (Cat. No.: G108397, Shanghai Aladdin Biochemical Technology Co., Ltd.) was completely dissolved in 50 mL of 1× PBS (pH 7.4) by stirring at 55 °C. The acidity of PBS was adjusted by adding sodium hydroxide (Cat. No.: 30620, Sigma-Aldrich). 700 μL of methacrylic anhydride (Cat. No.: L14357, Alfa Aesar) was dropped into the gelatin solution at a rate of around 0.5 drop/s while vigorously stirring. After a 3 h reaction, the mixture was diluted with 100 mL of 55 °C 1× PBS to slow down the reaction. The mixture was then transferred into a dialysis bag with a molecular weight cutoff of 12–14 kDa and dialyzed against DI water for 6 days at 40 °C under stirring. The DI water was refreshed every 8–12 h. The dialyzed solution was transferred to a −80 °C refrigerator for prefreezing overnight and then lyophilized for 4 days. The foamy GelMA was harvested and stored at −80 °C. To prepare the precursor, GelMA, gelatin, and lithium phenyl-2,4,6-trimethylbenzoylphosphinate (LAP) (Cat. No.: L157759, Shanghai Aladdin Biochemical Technology Co., Ltd.) were dissolved in PBS separately and then mixed to obtain the precursor solution for fabrication of the hydrogel. The final concentration of LAP was 0.6% (w/v). The final concentration of 4%, 8%, and 16% GelMA was employed to obtain different Young’s moduli of hydrogels. Gelatin was supplemented, as indicated in Table S1.

### 2.2 Microtunnel Fabrication with the UV-Patterning System

The DMD system was established to generate microfeatured UV patterns for printing the hydrogel structures. Specifically, the light-emitting diode UV beam (Opsytec Dr. Gröbel GmbH) was collimated using the combination of an aperture and a converging lens, then reflected and patterned via the programmed DMD board (DLPLCR70EVM, Texas Instruments, USA), and finally scaled down and transmitted onto the samples through a microscope platform (ECLIPSE Ts2R, Nikon) (Figure S1a,b). The working wavelength and reflectivity/transmittance of the mirrors (300 nm-750 nm, reflectivity >99%) and lens (245–400 nm, transmittance > 99.5%) used for our system were compatible with the UV light source employed (365 nm). Measured by a light intensity sensor, the final output UV pattern had a stable power of 650 mW/cm^2^.

A PDMS microfluidic chip was fabricated to hold the printed hydrogel channels and the cells migrating inside them using the classical protocols.^19^ Briefly, the SU8-2015 photoresist was lithographed on the silicon wafer to obtain the mold. The PDMS polymer and cross-linker were mixed in a ratio of 10:1 (w/w), degassed in vacuum, and then poured onto the mold and cured at 80 °C for 2 h. The molded PDMS was lifted off, punctured for the inlet and outlet holes, and finally bonded onto the glass slide after plasma treatment. The obtained PDMS chips had a channel height of around 20 μm, which is slightly larger than the size of the tested cancer cells, MDA-MB-231 (abbreviated as MB231).

The GelMA precursor was injected into the PDMS chip. As demonstrated in Figure 1b, stripe-shaped UV patterns were projected onto the precursor successively to produce a hydrogel channel with a varied gap distance. 9 μm wide UV stripes were employed in this study to obtain a stable structure. The exposure time for each UV stripe was 10 s. After printing, the hydrogel-loaded chips were warmed at 37 °C for 30 min. The unexposed precursor was then rinsed out with the warmed PBS to obtain the hydrogel channels ready for loading of the cells.

### 2.3 Cell Experiments

MB231 cell lines, purchased from ATCC, were cultured using the complete medium containing DMEM (Cat. No.: 11965092, Gibco) supplemented with 10% (v/v) fetal bovine serum (Cat. No.: A5256701, Gibco) and 1% (v/v) penicillin–streptomycin (Cat. No.: 15140122, Gibco) and incubated in 5% CO_2_ at 37 °C. Human umbilical vein endothelial cells (HUVECs), purchased from Sigma-Aldrich, were cultured using the human large vessel endothelial cell basal medium (Cat. No.: M200500, Gibco) with the same supplements. Cells were digested with 0.25% trypsin-EDTA (Cat. No. 25200072, Gibco) for harvesting. Cells were resuspended in the fresh medium and then injected into the migration chip using a microsyringe. The cell-loaded chip was immersed in the medium and incubated for subsequent testing. Cell migration was recorded under a bright field using a living cell platform integrated in an inverted microscope system. After living cell experiments, the samples were fixed with 4% paraformaldehyde (Cat. No.: 281692, Santa Cruz) and permeabilized with 0.1% Triton X-100 (Cat. No.: T109026, Shanghai Aladdin Biochemical Technology Co., Ltd.) at room temperature for 20 min and then washed with PBS for fluorescence staining.

### 2.4 Characterization of Hydrogel Tunnels

Young’s modulus of the hydrogels was measured by atomic force microscopy (AFM; JPK NanoWizard II, Bruker). The samples were indented by a bead-attached tip under contact mode. The spring of the used tip was 0.03 N/m (Cat. No.: ARROW-TL1–50, NanoWorld). To attach a 10-μm-diameter polystyrene bead (Cat. No.: PS07001, Bangs Laboratories, Inc.) onto the tip, the tip end was dipped in a mixture of epoxy glue, then compressed onto the bead by AFM, and held for 5 min. The tip attached with a bead was then lifted and stored overnight for complete curing. The force curve data were fitted using the Hertz model to calculate the Young’s modulus. The actual gap distance of the printed channel walls was measured after swelling. The gel samples were stained with 0.01% (w/v) fluorescein isothiocyanate (Cat. No.: F106837, Shanghai Aladdin Biochemical Technology Co., Ltd.) for 2 h and then rinsed with PBS. The fluorescence images were analyzed using ImageJ software.

### 2.5 Fluorescence Staining and 3D Measurement

The cells migrating inside the channels were picked up for characterization. The fixed samples were stained with 10 μg/mL Hoechst33342 (Cat. No.: H3570, Invitrogen) and 165 nM 647-conjugated phalloidin (Cat. No.: A22287, Invitrogen) in PBS at room temperature for 45 min and then rinsed with fresh PBS. The nucleus was then imaged by a laser scanning confocal microscope to achieve 3D information. Each stack image was analyzed, and the nuclear region in it was identified and extracted by using an edge-detection algorithm in MATLAB. The 3D structure of the nucleus was then reconstructed, and its volume could thus be calculated.

Nonmuscle myosin heavy-chain II-A was stained and imaged to quantify the expression level of nonmuscle tractive myosin in cells. The fixed samples were blocked with 5% normal goat serum (NGS) (Cat. No.: SL038, Solarbio Life Science) at room temperature for 1 h. Then, the blocking serum was replaced with 1:200 diluted myosin primary antibody (Cat. No.: 909801, BioLegend) in 5% NGS, and the samples were incubated at 4 °C overnight. After that, the samples were rinsed with fresh PBS and then stained with 1:1000 diluted 488-conjugated secondary antibody (Cat. No.: A32731, Invitrogen) in PBS at room temperature for 1 h. The samples were then rinsed with PBS. The myosin in the cells was also imaged via a confocal microscope. The expression levels of intracellular myosin II were quantified by summing the signal detected from each stack image. The data of confined migration groups were normalized by dividing the average expression value of the cells cultured on the glass surface control.

### 2.6 Quantification and Statistical Analysis

Data are presented as the mean ± SD. Statistical significance was estimated, and P-values were calculated by performing the one-way analysis of variance with Tukey’s post hoc test. All experiments were repeated at least three times.

## 3 Results and Discussion

### 3.1 Fabrication of Microtunnels with Customized Stiffness for Cell Migration Study

The microtunnels were fabricated by printing the array of hydrogel walls with a predefined height of 20 μm (see the printing principle in Figure 1b, printed hydrogel tunnels in Figure 1c, 3D confocal imaging of the tunnel wall in Figure 1d, and the actual photo of the PDMS chamber in Figure S1c). In this setup, the resulting tunnel width can be adjusted by changing the gap distance between two individual UV stripes (Figure 1b,c). Specifically, by measuring the actual tunnel width after swelling of the hydrogel walls from fluorescence images (refer to the inset in Figures 1b and S1d), a linear relationship between the designed gap distance of UV stripes and the resulting tunnel width (Figure S1d,e) was observed. Tunnels with a width of around 5 and 8 μm were used in this study for examining the confined movement of MB231 cells that have a diameter of ∼17.5 μm (Figure S1f). Specifically, cells were allowed to crawl into and out of the 100 μm long tunnel (Figure 1a). Our AFM measurement showed that the wall modulus increased from 3.21 to 8.26 kPa and then to 17.84 kPa when the GelMA concentration was varied from 4, 8, and 16% (Figure S1g). Note that gelatin was supplemented to serve as the cell-adhesive polymer. To make sure that the affinity between cells and hydrogels does not change (due to the dissolving of the hydrogel in warm solution) significantly among different groups, we have monitored the weight change of GelMA-Gelatin mixtures under body temperature incubation. Specifically, hydrogel samples were swelled at 37 °C for 24 h and then lyophilized for weight measurement. In comparison, hydrogels in the control group were lyophilized without undergoing swelling at 37 °C. Our results showed that, when compared to the control group, 24 h incubation resulted in 25, 17, and 5% weight loss for “4%+12%”, “8%+8%”, and “16%+0%” GelMA-Gelatin hydrogels, respectively (Figure S1h). Since the confined migration of cells all occurred within 24 h in our experiment, such weight loss is acceptable because recent studies have shown that a gelatin polymer with a concentration of around 5–10% would be enough for supporting/facilitating cell adhesion and migration.^20,21^ For simplicity, the three hydrogels are referred to as 4, 8, and 16% GelMA hydrogels, respectively, in subsequent sections.

### 3.2 Biphasic Dependence of Cell Speed on the Stiffness of the Confining Wall

**Figure 2:**
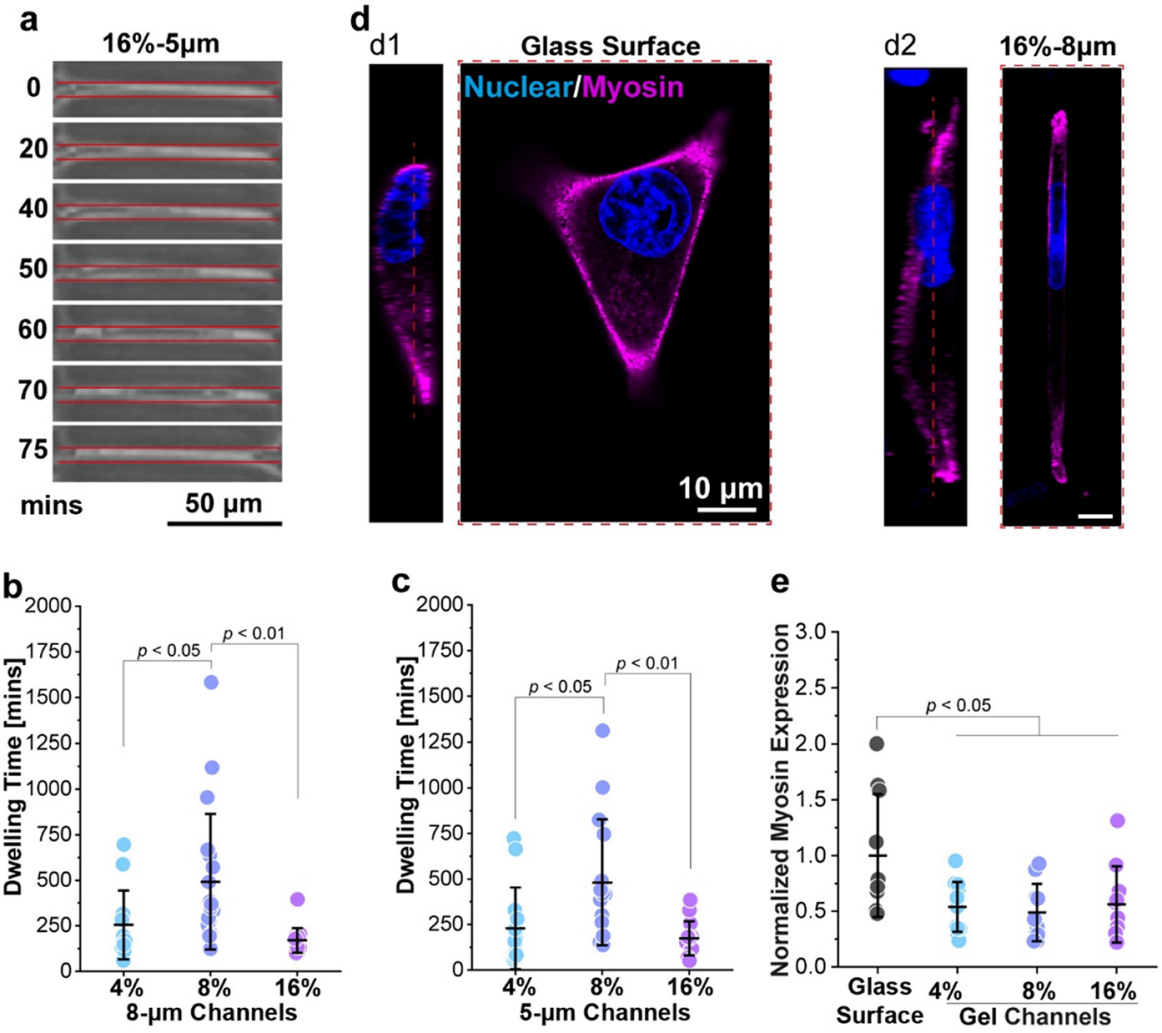
Rigidity-dependent migration of cells under confinement. (a) Bright field recordings of an MB231 cell migrating into and out of a 5-μm microtunnel fabricated from 16% GelMA, where the tunnel wall surface was indicated by the red line. (b) Dwelling time of cells inside the 8 μm tunnel fabricated with 4% (*n* = 13), 8% (*n* = 19), and 16% (*n* = 16) GelMA. (c) Dwelling time of cells inside the 5-μm tunnel fabricated with 4% (*n* = 13), 8% (*n* = 15), and 16% (*n* = 15) GelMA. (d) Fluorescence images showing the nuclear morphology (blue) and intracellular myosin (purple) distribution in the cell cultured on glass (see d1) or trapped in the 8 μm microtunnel (see d2) fabricated with 16% GelMA. (e) Comparison of myosin expression level in cells cultured on glass (*n* = 10) or trapped in 8 μm microtunnels fabricated from 4% (*n* = 12), 8% (*n* = 10), and 16% (*n* = 10) GelMA. Data dots in panels (b), (c), and (e) represent the measured value, and the corresponding data lines are presented as the mean ± SD. *P*-values were calculated by performing the one-way analysis of variance with Tukey’s post hoc test.

MB231 cells migrated into the tunnel by squeezing and elongating themselves and then were trapped inside for several hours until they completely exited (Figure 2a). Interestingly, the average time for the cell to pass through the 100 μm long confined tunnel exhibited a biphasic trend as the wall modulus increased from 3.21 to 17.84 kPa. In particular, the longest dwelling time (or equivalently the lowest cell speed) was achieved in both 8 and 5 μm tunnels when the tunnel wall was formed with the hydrogel containing 8% GelMA, i.e., when the stiffness of the confining boundary is at an intermediate level (Figure 2b,c, Videos S1 and S2). This trend is totally different from the well-known durotaxis behavior of cells migrating on a 2D substrate, indicating that a new rigidity-dependent regulation mechanism has been introduced by the confinement. Note that we have repeated the same set of experiments on normal HUVECs. Interestingly, a similar biphasic trend was observed (see Figure S2 and Video S3), although HUVECs appeared to move much slower than MB231 cells.

We then examined the distribution and expression levels of myosin II, a key traction-related motor protein, in migrating cells. Interestingly, myosin II was found to be expressed along the cell periphery and aggregated at the protrusions of cells (see d1 in Figure 2d), moving on the glass surface (as a control). In comparison, highly concentrated myosin II was observed at two ends of the cell migrating in the tunnel (see d2 in Figure 2d). Surprisingly, the total quantity of myosin II in cells confined within the tunnel is only half of that in cells cultured on the glass surface (Figure 2e), suggesting attenuated traction generated by cells under confinement. Moreover, no significant difference in myosin expression was found among the tunnel groups with different wall stiffnesses (Figure 2e). This indicates that myosin activity is unlikely to be the reason behind the biphasic trend of cell speed against confining environment rigidity observed here.

### 3.3 Reduced Nuclear Volume under Stiffer Confinement

**Figure 3:**
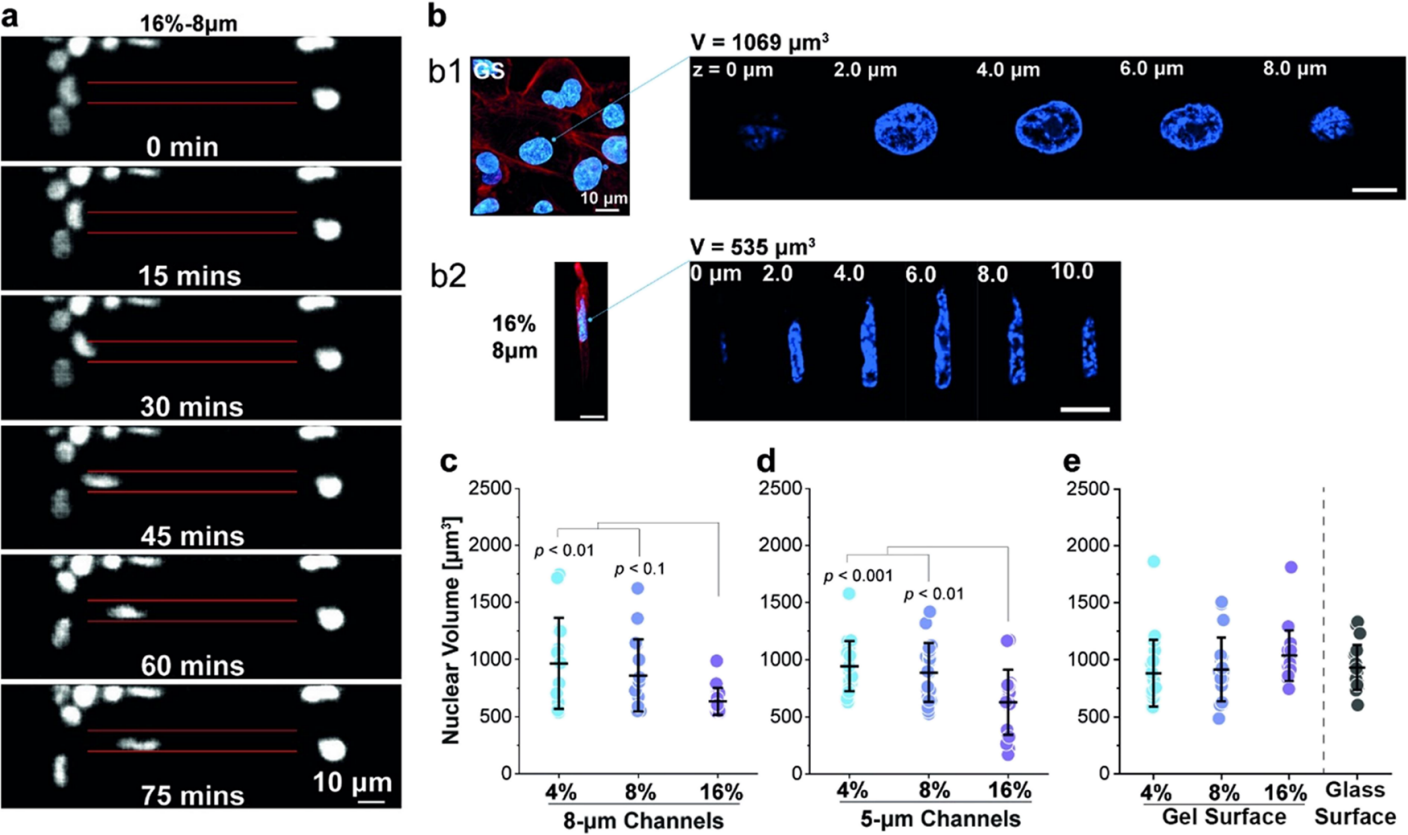
Significant nuclear deformation of cells under confinement. (a) Fluorescence recording showing the nuclear morphology evolution of an MB231 cell migrating in the 8 μm microtunnel (red line). (b) Representative fluorescence images of cells (red: actin; blue: nucleus) cultured on glass (GS) (see b1) or trapped in the microtunnel (see b2). (c–e) Nuclear volume can be estimated from the stacked confocal images. Dependence of nuclear volume of cells on the rigidity of the wall of (c) 8 μm or (d) 5 μm tunnels, and (e) surface. *n* = 20 for each group shown in panels (c–e). Data dots in panels (c–e) represent the measured value, and the corresponding data lines are presented as the mean ± SD. *P*-values were calculated by performing the one-way analysis of variance with Tukey’s post hoc test.

Nucleus is the largest and stiffest cell organelle, whose response largely determines the deformability of the cell during its migration inside our microtunnels (Figure 3a). Indeed, a highly elongated nuclear morphology inside the tunnel was observed by confocal imaging (see b2 in Figure 3b), in direct contrast to the pancake-like nucleus in cells migrating on the glass surface (see b1 in Figure 3b). From the stacked confocal images, the nuclear volumes of confined MB231 cells were also measured. Interestingly, such volume was found to decrease as the stiffness of the tunnel wall increases (Figure 3b–d). In comparison, no detectable difference in the nuclear volume was observed when cells were cultured on the glass or hydrogel surfaces with the same rigidities as those of the tunnel walls (Figure 3e). The highly deformed nucleus indicates the presence of significant contact force between the cell and the tunnel, which in turn could generate friction against the movement of cells.

### 3.4 Pulling Race Model for the Confined Cell Migration

**Figure 4:**
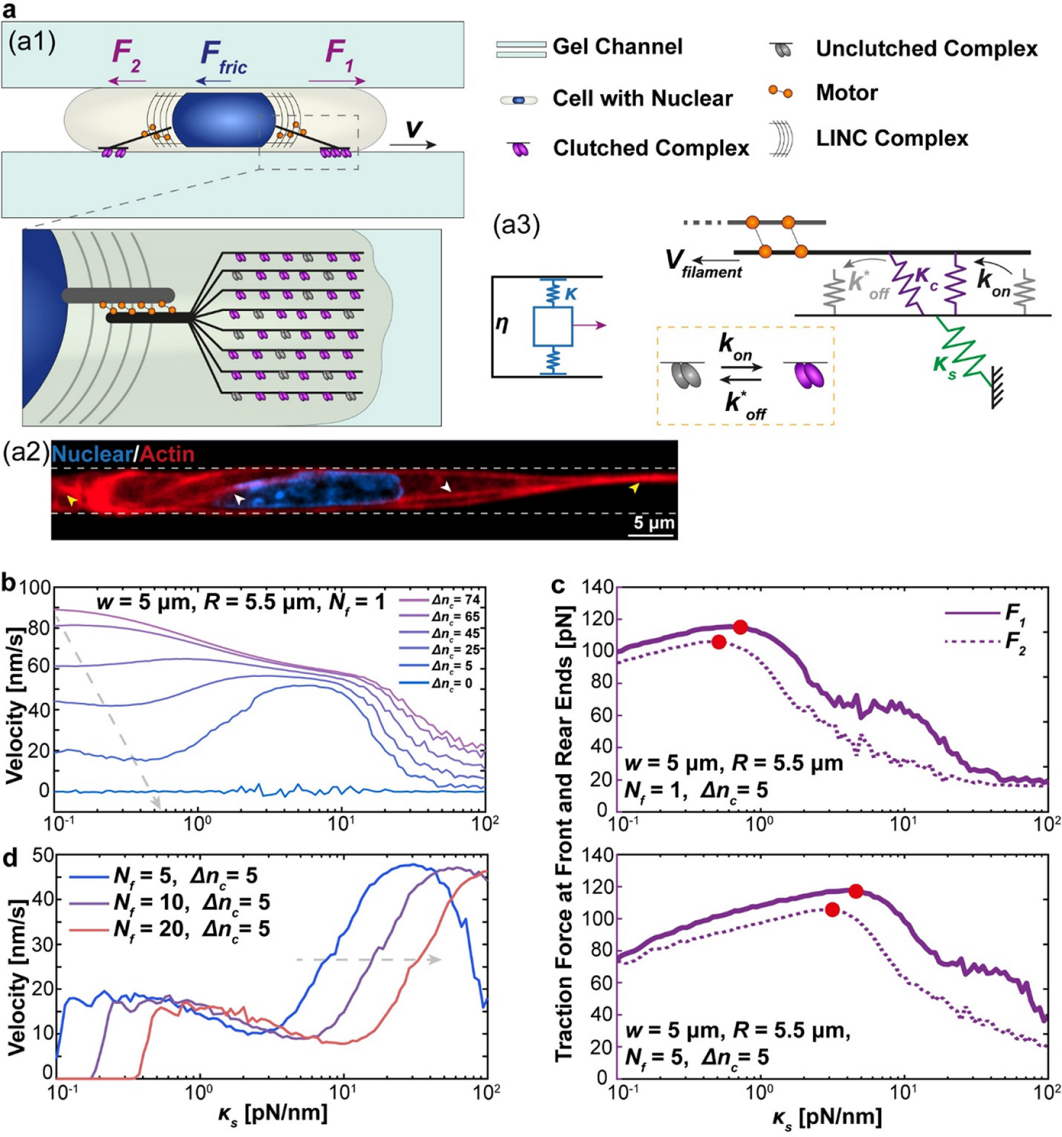
Pulling race model explaining the observed biphasic dependence of cell speed on the stiffness of the confining tunnel wall. (a) Schematic illustrating that the cell movement is driven by the biased force generation at its two ends and resisted by the friction between the nucleus and the tunnel wall (see a1). The nucleus is assumed to connect to an array of actin fibers and therefore the tunnel wall via molecular clutches on both ends of the cell (see the inset in a1). Fluorescence image showing the nuclear morphology (blue) and actin (red) distribution of an MB231 cell inside the 5 μm microtunnel (white dash lines) is given in a2, where actin aggregation and stress fiber formation are indicated by the yellow and white arrows, respectively. The equivalent mechanical model and illustration of stochastic clutch association/disengaging are shown in a3. (b) Simulated velocity of cells as a function of the wall stiffness (κ_s_) and the clutch number difference (Δ*n*_c_) between the two ends of each actin fiber. Note that the total clutch number difference between the front and rear cell ends is Δ*n*_c_ × *N*_f_, with *N*_f_ representing the total number of actin fibers. The clutch number at the front end of the cell was fixed as *n*_c,front_ = 75 here. Interestingly, the cell speed will reach a local minimum at intermediate wall stiffness when Δ*n*_c_ is relatively small. (c) Simulated forces generated at the front and rear ends of the cell as functions of tunnel wall stiffness. The onset of adhesion-to-slippage transition was indicated by red dots. (d) Stiffness leading to locally minimized cell velocity shifts to larger values when the number of actin fibers (or equivalently the number of total clutches) increases.

Based on the aforementioned observations, we proceed by developing a motor-clutch-based pulling race model to explain the confined migration of cells. Specifically, to simplify the analysis, the cell is represented by an elastic nucleus (originally having a spherical shape) connected to multiple tractors on both sides; see Figure 4a. Each tractor consists of actin fibers and myosin motors and is connected to the nucleus and the tunnel wall via the LINC (linker of nucleoskeleton and cytoskeleton) complex and integrin-based molecular clutches (see a1 and a2 in Figure 4a), respectively. Due to myosin contraction, forces will be generated within the tractor, which then can be transmitted to the cell nucleus and drive its movement.

To estimate the force generated by the tractor, we use the well-known motor-clutch model, where the actomyosin fiber is assumed to be connected to the deformable tunnel wall via a number of transient motor clutches.^11,22^ Contraction of myosin will drive the actin fibers to slide with respect to the wall, a motion that will be resisted by engaged clutches, which then in turn induce a force on the cell. We proceed by assuming multiple actin fibers (each consisting of *n*_c_ clutches) exist in the front and rear ends of the cell that connect the tunnel wall with the cell nucleus (see a1 in Figure 4a). Under such circumstances, the total force *F* generated at the front/rear end of the cell can be expressed as
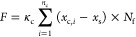
1where *N*_f_ is the total number of actin fibers, *x*_c,*i*_ represents displacement of the *i*-th clutch and we have *x*_c,*i*_ = *x*_s_ for the disengaged state (see a3 in Figure 4a), *x*_s_ stands for the displacement of the substrate taking the form derived from the force balancing relation *F* = *x*_s_ × κ_s_
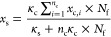
2Here, κ_s_ represents the stiffness of the substrate and κ_c_ is the effective spring constant of the engaged clutch. The transition of clutches from the engaged to the disengaged state (and vice versa) can occur in a stochastic manner. In particular, following the Bell model,^23^ the average dissociation rate *k*_off_ for an engaged clutch is assumed to increase exponentially with the force *f* acting on it as
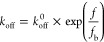
3where *k*_off_^0^ is a constant rate and *f*_b_ represents the force scale associated with the breakage of the clutch. On the other hand, every disengaged clutch can rebind with a constant rate *k*_on_. Finally, the contraction-induced velocity *v*_f_ of actin fibers (i.e., the actin retrograde flow velocity) is given by^24,25^
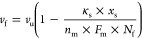
4where *n*_m_ is the number of myosins acting on each actin fiber, *F*_m_ represents the maximum contraction force a myosin motor can generate, and *v*_u_ is the maximum sliding velocity of myosin when there is no resistance force.

If *F*_1_ and *F*_2_ represent the total force generated at the front and rear end of the cell, respectively, then their difference (*F*_1_ – *F*_2_) will cause the cell to move inside the tunnel, as well as be balanced by the viscous force from the tunnel against such movement, that is
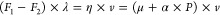
5where *v* is the migration speed of the cell and η stands for the frictional coefficient between the cell and the tunnel. Note that here, η is assumed to contain two parts: one representing the constant friction coefficient μ from the bottom surface and the surrounding water, and the other α × *P* is assumed to be proportional to the compression force *P* between the cell nucleus and the tunnel wall. In addition, a geometric factor λ is introduced to the 1D description here to reflect the fact that actin fibers will become more aligned with the tunnel as its width *w* decreases, effectively resulting in a higher component of the traction forces that can be used to drive the movement of cells along the tunnel. Here, for simplicity, this factor is taken as , where *w*_0_ (chosen as 10 μm) is a characteristic width at which actin fibers have totally random orientations and consequently cannot generate effective forces for the cell to move.

Next, Hertz contact theory is used to estimate the contact force between the cell nucleus and the tunnel wall as
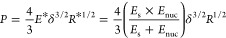
6where *R* and δ represent the initial radius and compression distance of the cell nucleus, and *E*_s_ and *E*_nuc_ refer to the elastic moduli of the tunnel wall and nuclei, respectively. Finally, given that cells were observed to move in the same direction for most of the time in our experiment, a small difference in the number of clutches per actin fiber, Δ*n*_c_, at the leading and trailing ends of the cell was assumed. The values of parameters used are shown in Table S2. Monte Carlo simulations were conducted to capture the random breakage/re-engagement of molecular clutches connecting each actin fiber with the tunnel wall,^22^ calculate the force generated by the tractor, and then finally simulate the movement of cells within the tunnel (see Figure S3 and Supporting Information for details).

Interestingly, a biphasic relationship between the wall stiffness and the migration speed of cells, consistent with our experimental observations, was indeed predicted from this simple model, as long as the difference Δ*n*_c_ in clutch numbers at the front and rear ends of the cell is within a relatively small range. However, the biphasic trend disappears when the Δ*n*_c_ becomes large (say when Δ*n*_c_ > 25), as illustrated in Figure 4b. On the other hand, the average cell speed will decrease to almost zero when both ends have the same number of clutches (i.e., when Δ*n*_c_ ∼ 0).

To understand the physical mechanism behind this, let us recall that an adhesion-to-slippage transition (AST) will take place in the original motor-clutch model as the substrate stiffness increases,^11^ leading to a suddenly dropped traction force. Before AST occurs, cell traction force increases via stiffness-enhanced adhesion, and the growth of net force (*F*_1_ – *F*_2_) will saturate quickly due to the small difference in clutch number at the front and rear ends of the cell (Figure S4). Meanwhile, the stiffness-dependent frictional coefficient grows rapidly (Figure S4), resulting in a reduced migrating speed at relatively intermediate stiffness regimes. Importantly, the critical ECM stiffness for triggering AST depends on the number of clutches, with more clutches resulting in a larger transition stiffness (Figure 4c). Consequently, if the difference in the clutch numbers between the two cell ends is relatively small, then within a narrow ECM stiffness range, AST may already take place in the rear end but not yet occur at the front end of the cell (Figure 4c,d). This will result in an exploding increase of net traction force, and meanwhile, the frictional coefficient is saturated at a high stiffness regime, giving rise to an elevated speed. All these result in a V-shaped biphasic trend of migrating speed of cells in the confining tunnel.

### 3.5 Nuclear Deformation-Induced Resistance against Confined Cell Migration

**Figure 5:**
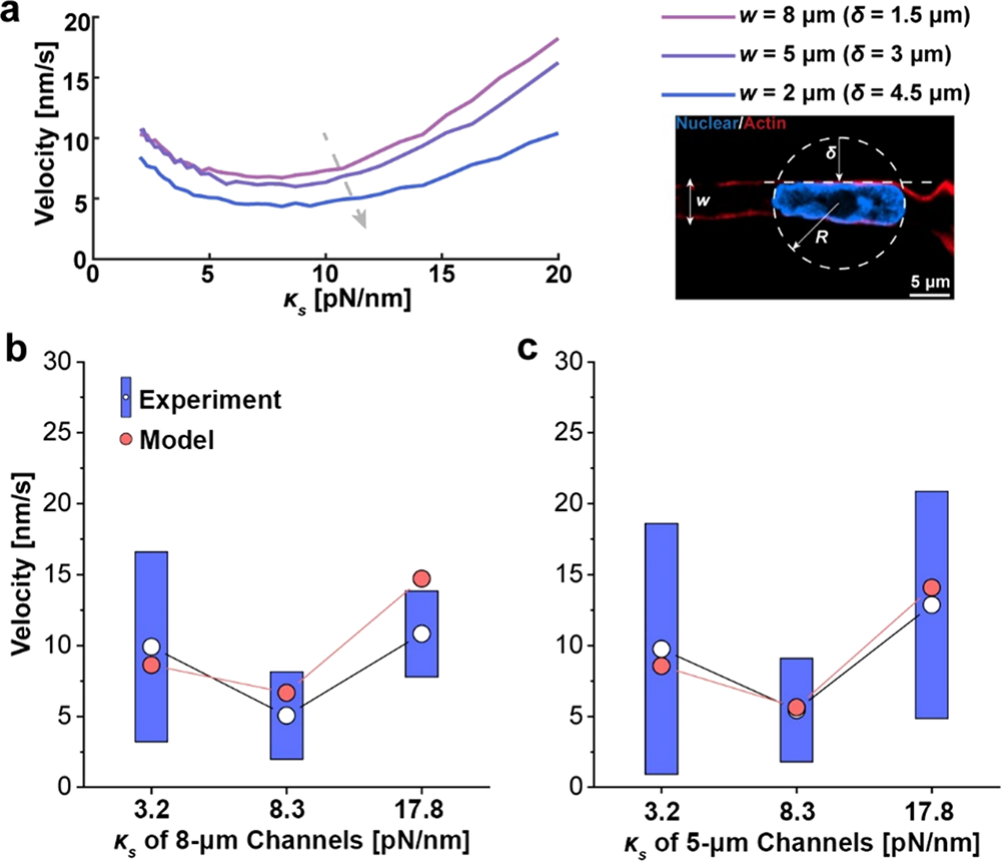
Comparison between model predictions and experimental data. (a) Simulated cell velocity as a function of both the tunnel width and wall stiffness. Results shown here are based on 20 million steps of Monte Carlo simulations according to our model where the number of actin fibers was chosen to be 15. The inset on the right illustrates how nuclear deformation inside the microtunnels was calculated. Basically, the compression δ exerted on the nucleus was estimated as δ = *R* – *w*/2, with *R* and *w* representing the initial radius of the cell nucleus and tunnel width, respectively. (b,c) Using the same set of parameters shown in Table S2, the predicted cell speed matches well with our measured data for both (b) the 8 μm and (c) 5 μm tunnels with different wall stiffness. Note that 4, 8, and 16% GelMA gel correspond to a wall stiffness of ∼3.2, 8.3, and 17.8 pN/nm, respectively.

The effect of nuclear deformation on the migration speed of cells was represented by a contact force-dependent friction coefficient (eq 5). Evidently, a decreasing tunnel width (e.g., from 8 to 2 μm) will cause increasing nuclear deformation and therefore lead to higher resistance against cell movement (Figure 5a), which is totally different from the migration of cells on a 2D surface. Experimentally, the cell nucleus was found to be a few times stiffer than the cell body.^26,27^ Given the stiffness of MB231 cells (used in this study) is believed to be in range of ∼0.5–1 kPa,^28^ a nuclear modulus value of 1.5 kPa was adopted here. The following relation
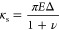
7was employed to convert the Young’s modulus, *E*, into substrate stiffness, κ_s_.^11^ Here, Δ is the characteristic distance (typically less than one micron) away from the cell edge within which the strain on the substrate exerted by the cell becomes detectable. ν is the Poisson ratio of the hydrogel (ν = 0.3–0.5). Based on these values, an approximate mapping of 1 kPa in modulus to 1 pN/nm in stiffness was established and employed in this study. Interestingly, the simulated cell speed from our model quantitatively matches well with the experimental data (Figure 5b,c), where a local minimum of cell velocity was achieved at intermediate wall stiffness for both 8 and 5 μm tunnels.

## 4 Conclusions

A biphasic trend of the cell speed with the increasing stiffness of the wall of microtunnels confining the cell was found in this study, resulting in a local minimum of the cell velocity at an intermediate wall modulus of around 10 kPa. Interestingly, more significant nuclear deformation was observed as the tunnel wall became stiffer, whereas no detectable change in the expression level of myosin in cells was found. Based on this information, a motor-clutch-based pulling race model was proposed, where cellular movement is caused by uneven traction force generation at the front and rear ends of the cell and resisted by nuclear-wall contact-induced friction. Choosing realistic parameter values, a biphasic trend of the cell speed (against tunnel wall stiffness) was indeed predicted by the model, in quantitative agreement with our measurement data. It must be pointed out that, in reality, the dynamics of actin assembly, disassembly,^29^ and stiffness-dependent morphology and anisotropy of cells^30^ could also play a role in how cells migrate in confined microenvironment with distinct mechanical properties. However, these issues are beyond the scope of this study and are left for future investigations.

Finally, we want to emphasize that the biphasic trend of cell velocity discovered here is caused by both rigidity-dependent traction generation and nuclear deformation and, therefore, is totally different from the well-known durotaxis or negative durotaxis behavior of cells. In addition, unlike most previous studies where very stiff materials (such as PDMS) were used to fabricate the confining microchannels, the moduli of tunnel walls adopted here varied from a few to a few tens of kPa (comparable to that for normal or fibrotic tissues in cancer patients); therefore, our findings could have direct implications on how processes such as cancer metastasis and embryo development progress in vivo.

## Supporting Information Available

MB231 cell migrating in the 8 μm tunnel (Video S1) (AVI)
MB231 cell migrating in the 5 μm tunnel (Video S2) (AVI)
HUVECs migrating in the 8 μm tunnel (Video S3) (AVI)
Demonstration of the DMD system, chip chamber, printed hydrogel, size of tunnels and cells (Figure S1); dwelling time of HUVECs inside the 8 μm tunnel fabricated with 4, 8, and 16% GelMA (Figure S2); illustration of the steps involved in our Monte Carlo simulation (Figure S3); simulated forces generated at the front and rear ends of the cell, as well as the simulated frictional coefficient (Figure S4); illustration of synthesizing the GelMA polymer and fabricating hydrogels via the UV-induced photo-cross-linking reaction (Figure S5); formula of GelMA-Gelatin hydrogels for microtunnel fabrication (Table S1); and parameters adopted in our model (Table S2) (PDF)

## Supplementary Material

am5c03048_si_001.avi

am5c03048_si_002.avi

am5c03048_si_003.avi
