# Supplementary material for: Nuclear Deformation and Stiffness-Dependent Traction Force Generation Dictate the Migration of Cells under Confinement
Source: ACS Appl Mater Interfaces. 2025 Apr 15;17(16):23731–9. doi: 10.1021/acsami.5c03048 (PMC12022946; doi:10.1021/acsami.5c03048)
Supplement: Supplementary file 4 — am5c03048_si_004.pdf [file am5c03048_si_004.pdf]

# Supporting Information

**Nuclear Deformation and Stiffness-dependent Traction Force Generation Dictate the Migration of Cells under Confinement**

Zheng Wang[1], Feng Xu[2], Di Wu[1], Wei Huang[1,3], Zhiqin Chu[2,3, *], Yuan Lin[1,3, *]

[1] Department of Mechanical Engineering, The University of Hong Kong, Central and Western District, Hong Kong SAR, 999077, China

[2] Department of Electrical & Electronic Engineering, The University of Hong Kong, Central and Western District, Hong Kong SAR, 999077, China

[3] Advanced Biomedical Instrumentation Centre, Hong Kong Science Park, Shatin, New Territories, Hong Kong SAR, 999077, China

[*] Correspondence: zqchu@eee.hku.hk (Z.C.); ylin@hku.hk (Y.L.).

**Supporting Information**
**Figures S1‑S5**
**Tables S1‑S2**
**Videos S1‑S3**
**References**

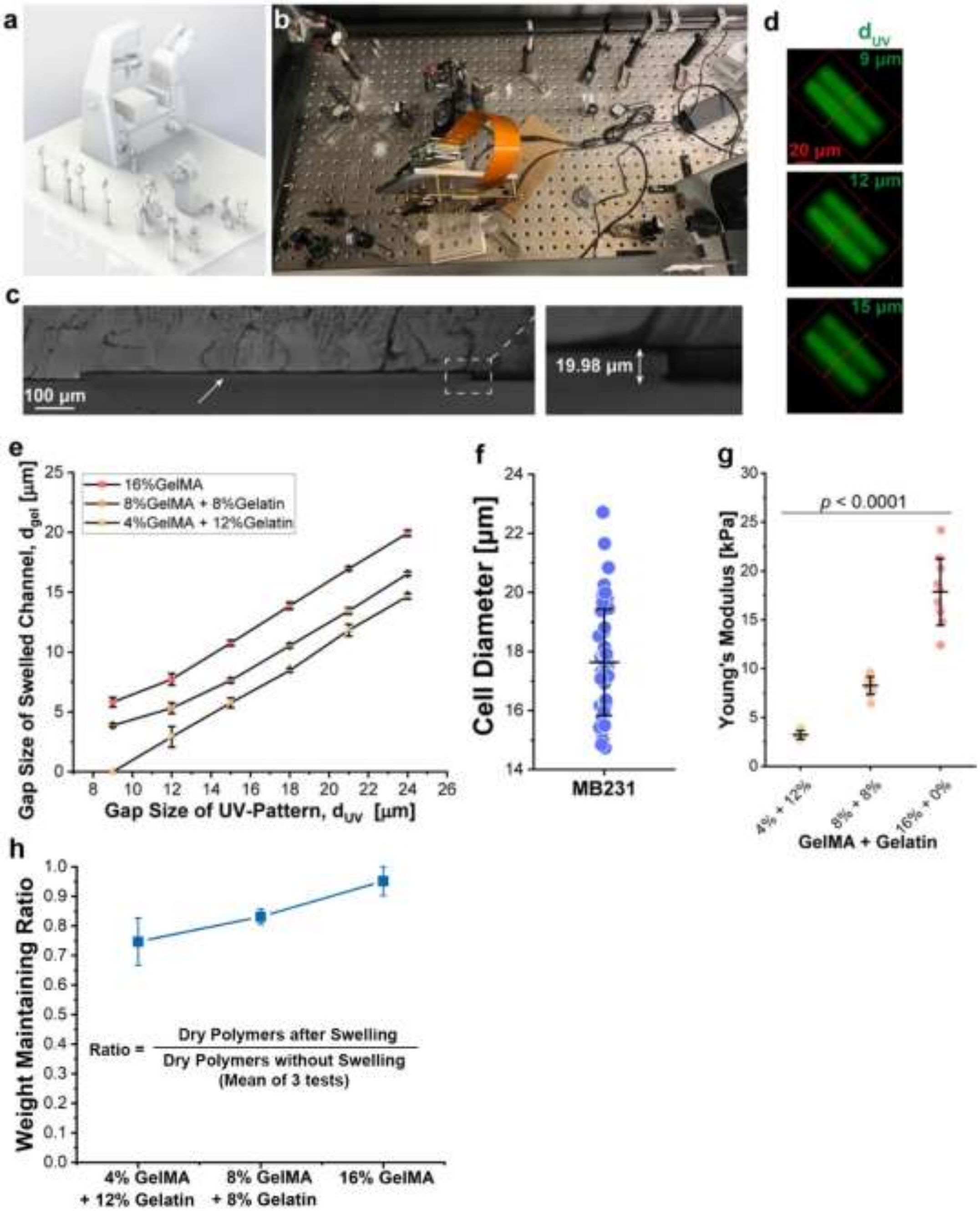


**Figure S1.** (a) 3D modeling and (b) actual photo of the DMD system. (c) Sideview of the PDMS micro-chamber with an estimated height of ~20 μm for housing the printed hydrogel micro-tunnels (also referring to the chip schematic shown in Figure 1B). (d) Fluorescent images of printed hydrogel micro-tunnels. (e) Measured gap size of hydrogel tunnels after swelling for different UV pattern exposure (n = 6 samples/group). (f) Measured size of cells (n = 50). (g) Measured Young's modulu of GelMA-Gelatin hydrogels with different GelMA/Gelatin concentrations (n = 10 samples/group). (h) Measured weight maintaining ratio of GelMA-Gelatin mixture after undergoing swelling at 37℃ for 24 hours (and then lyophilized for weight measurement) (n = 3 samples/group). Hydrogels in the control groups were lyophilized without undergoing swelling at 37℃.

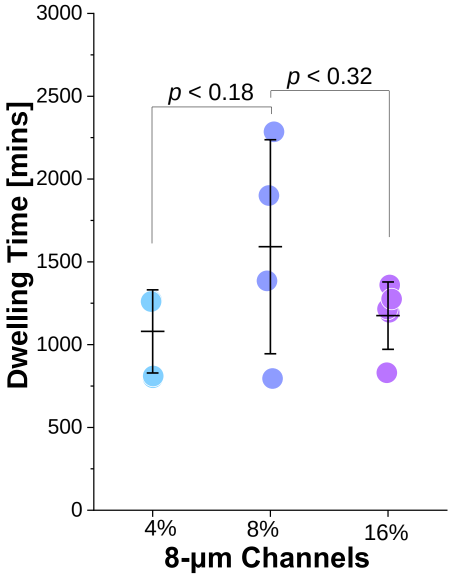


**Figure S2.** The dwelling time of HUVECs inside the 8-μm tunnel fabricated with 4%, 8% and 16% GelMA respectively (n = 5 cells/group)

**Table S1.** Formula of GelMA-Gelatin hydrogels for micro-tunnel fabrication

| Expected stiffness after cross-linking | %GelMa (w/v) | %Gelatin (w/v) | %GelMa + %Gelatin (w/v) |
|---|---|---|---|
| **Soft** | 4% | 12% | 16% |
| **Moderate** | 8% | 8% | 16% |
| **Stiff** | 16% | 0% | 16% |

**Table S2.** Parameters adopted in our model [1-2]

| **Parameters** | **symbols** | **value** |
|---|---|---|
| clutch spring constant | $\kappa_c$ | 5 pN/nm |
| number of motors on each fiber | $n_m$ | 75 |
| motor stall force | $F_m$ | 2 pN |
| motor unloaded velocity | $v_u$ | 120 nm/s |
| clutch unloaded off-rate | $k_{off}^0$ | 0.1 $s^{-1}$ |
| characteristic bond rupture force | $f_b$ | 2 pN |
| clutch on-rate | $k_{on}$ | 1 $s^{-1}$ |
| stiffness of tunnel wall | $\kappa_s$ | 0.1–100 pN/nm |
| | $E_s$ | 0.1–100 kPa |
| stiffness of cell nuclei | $E_{nuc}$ | 1.5 kPa |
| flow-induced frictional constant | $\mu$ | 0.3 pN·s/nm × $N_f$ |
| cell-tunnel frictional coefficient | $\alpha$ | 0.046 s/μm × $N_f$ |
| number of clutches on each fiber at front end of cell | $n_{c1}$ | 75 |
| number of clutches on each fiber at rear end of cell | $n_{c2}$ | 1–75 |
| number of traction fibers | $N_f$ | 1–20 |

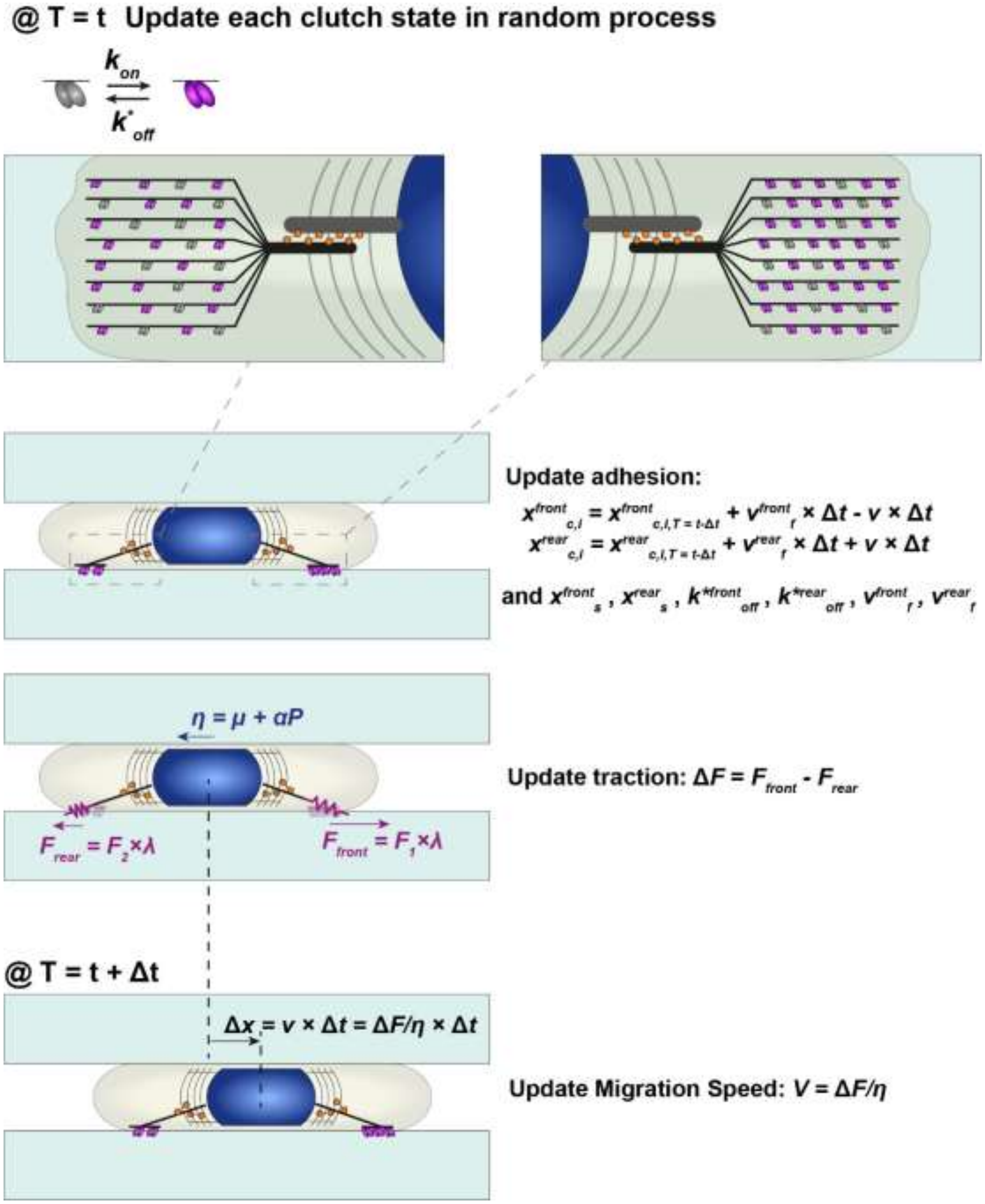


**Figure S3.** Illustration of the steps involved in our Monte Carlo simulation.

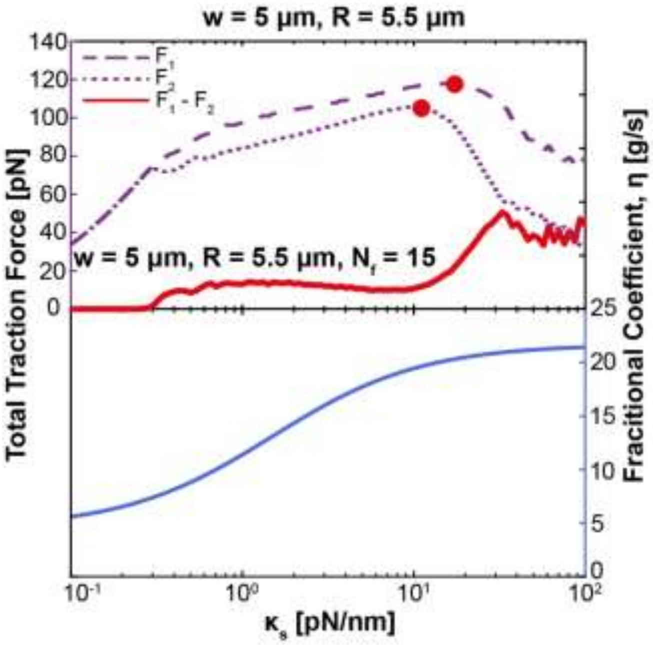


**Figure S4.** (Top) Simulated forces generated at the front and rear end of the cell, as well as the corresponding net force, as functions of the tunnel wall stiffness. The onset of adhesion-to-slippage transition was indicated by red dots. (Bottom) Simulated frictional coefficient as function of the tunnel wall stiffness.

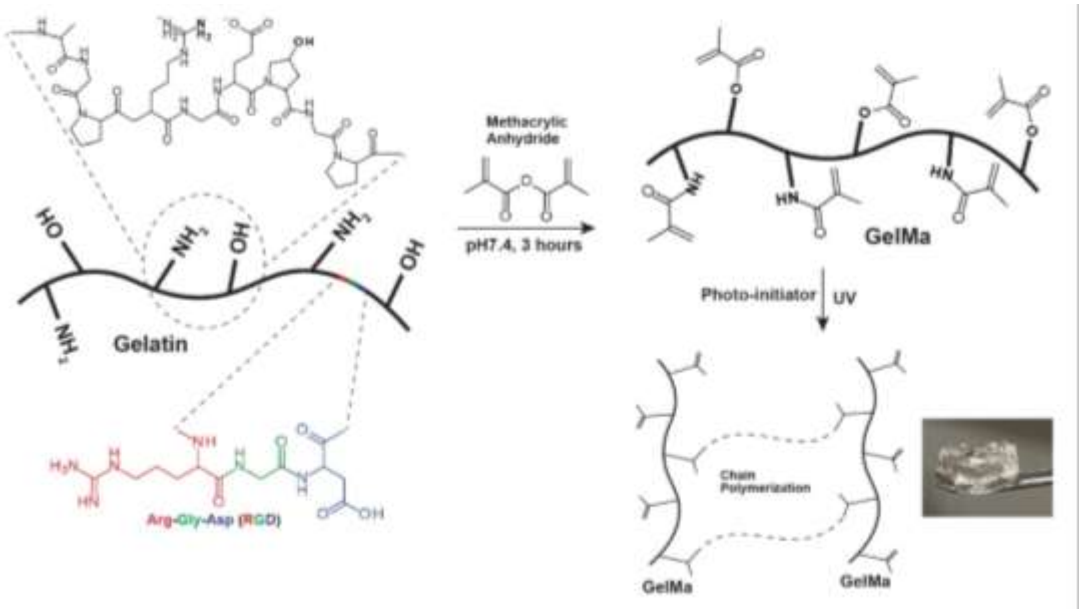


**Figure S5.** Illustration of synthesizing GelMA hydrogels via UV induced photo-cross-linking reaction.[3]

## References

(1) Bangasser, B. L.; Odde, D. J. Master equation-based analysis of a motor-clutch model for cell traction force. *Cellular and molecular bioengineering* **2013,** *6*, 449-459.

(2) Chan, C. E.; Odde, D. J. Traction dynamics of filopodia on compliant substrates. *Science* **2008,** *322* (5908), 1687-1691.
(3) Yue, K.; Trujillo-de Santiago, G.; Alvarez, M. M.; Tamayol, A.; Annabi, N.; Khademhosseini, A. Synthesis, properties, and biomedical applications of gelatin methacryloyl (GelMA) hydrogels. *Biomaterials* **2015,** *73*, 254-271.
